# The Effect of EMG biofeedback in female patients with stress urinary incontinence: a randomized sham controlled blinded clinical study

**DOI:** 10.1007/s11845-026-04374-3

**Published:** 2026-04-11

**Authors:** Basak Cigdem-Karacay, Esra Bayramoglu-Demirdogen, Naime Meric Konar, Selda Songur-Dagli, Figen Tuncay, Fatmanur-Aybala Kocak

**Affiliations:** 1 Department of Physical Medicine and Rehabilitation, Kirsehir Ahi Evran University Faculty of Medicine, Kirsehir, Turkey; 2 Kirsehir Ahi Evran University, Pilot University Coordinatorship of Health, Rectorship, Kirsehir, Turkey; 3 Department of Biostatistics and Medical Informatics, Faculty of Medicine, Bandirma Onyedi Eylul University, Balikesir, Turkey; 4 Department of Obstetrics and Gynecology, Kirsehir Ahi Evran University Faculty of Medicine, Kirsehir, Turkey; 5 Bandirma Onyedi Eylul University Faculty of Medicine, Balikesir, Turkey

**Keywords:** Pelvic Floor, Electromyography feedback, Quality of life, Urinary incontinence, Stress

## Abstract

**Aim:**

The effectiveness of EMG-BF in SUI has not yet been proven. This study aimed to the effectiveness of EMG-BF on pelvic floor muscle activity, pad test, severity of complaints, and quality of life in female patients with Stress Urinary Incontinence (SUI).

**Methods:**

In this randomized, placebo-controlled study, 60 women diagnosed with urinary incontinence were divided into three groups: pelvic floor muscle training (PFMT), EMG biofeedback (EMG-BF), and placebo EMG-BF. EMG-BF and Sham EMG-BF applications were applied 3 days a week for 8 weeks, for a total of 24 sessions The King’s Health Questionnaire (KHQ) and International Incontinence Questionnaire (ICIQ-SF), and 1-hour Ped Test were evaluated. Pelvic floor muscle activity was measured with EMG. All measurements were made at the beginning, at Week 8 and at Week 20.

**Results:**

No significant improvement was found in the control group in all parameters of ICQ_SF (p>0.05), while significant improvement was found in the EMG-BF and Sham EMG-BF groups (<0.001 for each). In the Pad test EMG-BF and Sham EMG-BF groups showed significant improvement compared to the control group (p<0.001). In EMG values ​​in EMG-BF and Sham EMG-BF groups (p<0.05), while no significant improvement was observed in the control group. (p>0.05)

**Conclusion:**

Adding EMG-BF therapy to PFMT exercises is effective on pelvic floor muscle activity, quality of life, frequency and amount of urinary incontinence. PFMT exercises alone did not have an effect on general health perception, role limitations due to incontinence, physical and social limitations, and personal relationships either.

**Clinical trial registration:**

After ethics committee approval, clinicaltrials.gov registration was made before patients were recruited into the study. (Clinical Trial NCT05366426)

**Supplementary Information:**

The online version contains supplementary material available at https://doi.org/10.1007/s11845-026-04374-3.

## Introduction

Stress Urinary Incontinence (SUI) is an extensive health issue affecting approximately 50% of women worldwide. SUI is defined as the sudden involuntary release of urine due to increased intra-abdominal pressure [1]. In the treatment of SUI, conservative treatment methods are primarily used for patients who do not have a surgical indication [1–3].

Lifestyle changes including weight loss, timed voiding, fluid restriction, behavioral changes, and pelvic floor muscle training (PFMT) are the first choice among conservative treatment options [1, 4]. PFMT is the basis of pelvic floor rehabilitation [5].

The PFMT can be applied alone or with the help of Electromyography Biofeedback (EMG-BF). For EMG-BF, internal electrodes placed the vagina or superficial electrodes placed in the perineal region are used. Through these electrodes, the patient is given visual and/or auditory feedback from a screen. Additionally, EMG-BF allows the therapist to monitor contractions myographically [6]. The EMG-BF assists the therapist in teaching the patient how to properly engage the pelvic floor muscles, especially in patients who are unable to isolate the pelvic floor muscles [5]. There are studies in the literature showing that rehabilitation with EMG-BF improves pelvic floor muscle *activation*, urinary incontinence and quality of life in female patients with SUI [5, 7, 8]. However, it has also been reported that there is no difference between performing PFMT with EMG-BF and without using EMG-BF in patients with SUI [9]. The effectiveness of EMG-BF in female patients with SUI has not yet been proven [10]. Current reviews report that more randomized controlled trials from different countries are needed to prove that adding EMG-BF to PFMT is an effective method [6, 11]. Additionally, as far as the authors are awere, there is no sham-controlled study in the literature evaluating the effectiveness of EMG-BF in pelvic rehabilitation. However, in the literature, the actual effectiveness of visual and auditory feedback in EMG-BF application was investigated using the sham method in studies evaluating the effectiveness of EMG-BF in neurological rehabilitation [12, 13], cardiac rehabilitation [14] and fibromyalgia treatment [15].

The aim of this study was to demonstrate the effectiveness of EMG-BF on pelvic floor muscle *activation*, pad test, severity of complaints, and quality of life in female patients with SUI using a sham application and a control group.

## Materials & methods

### Study design

This randomized controlled study was conducted with 60 patients within the Gynecology and Obstetrics outpatient and the Physical Medicine and Rehabilitation outpatient clinics of the Training and Redesignch Hospital between August 2022 and July 2024.

### Participants

**Fig. 1:**
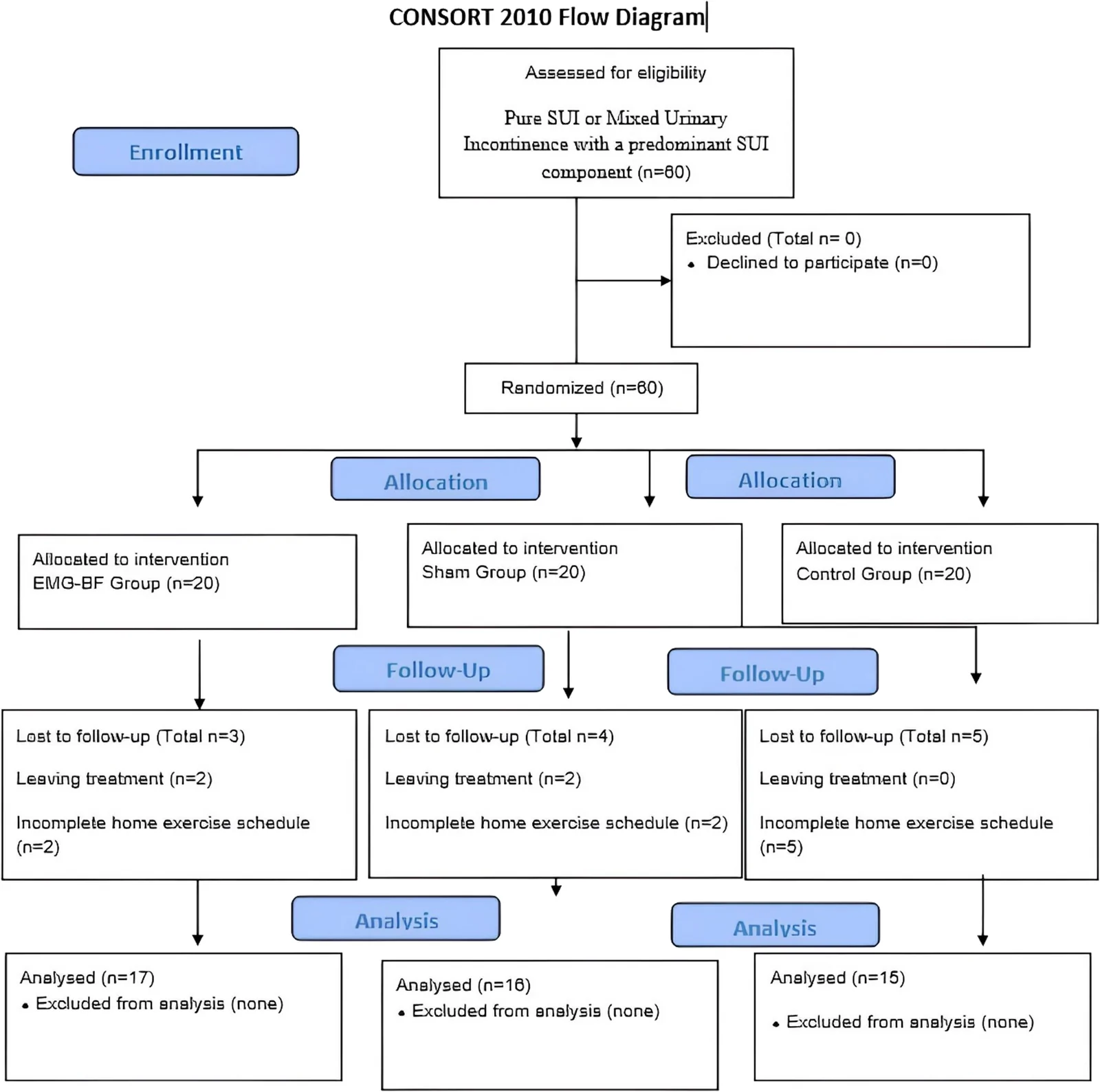
Flow Diagram

Female patients aged between 30 and 65 who were examined by a Gynecology and Obstetrics Specialist (W) and had a clinical diagnosis of Pure SUI or Mixed Urinary Incontinence with a predominant SUI component, and who were not planned for surgical intervention with this diagnosis, who reported the severity of urinary incontinence as 5 or above according to the 10-point visual analog scale, had not undergone pelvic rehabilitation in the last 3 months, and who agreed to participate in this study were referred to the Physical Medicine and Rehabilitation Clinic. The exclusion criteria for the study were determined as the presence of pure Urge or Urge-predominant Mixed Urinary Incontinence, change in medical treatment for urinary incontinence within the last 3 months, pregnancy, visual, auditory or cognitive dysfunction, performing high-intensity physical activity for at least 2 h per week, and the presence of serious systemic disease (diseases that prevent exercise-free application such as cardiovascular disease, chronic obstructive pulmonary disease, cerebrovascular accident, malignancy). (Fig. 1)

### Randomization and blinding

Patients were randomized into 3 groups, EMG-BF Group, Sham Group and Control Group, with the 1-1-1 method, according to the order of participation in the study. Randomization was performed by a different investigator (T) who was not involved in the intervention and evaluation. The investigator (X) who evaluated the outcome measures was unaware of the groups. The study data were recorded by coding the group names and sent to the person who performed the statistical analysis. Therefore, the researcher who performed the statistical analysis of the study (Z) was unaware of which group the data belonged to. When the study data was shared with the person performing the statistical analysis, care was taken to avoid including group names in the document. Thus, the researcher (Z) performing the statistical analysis of the study remained unaware of the group information.

### Interventions

#### EMG-BF program

The EMG-BF was applied with surface EMG electrodes placed in the perineal region in the hospital’s pelvic rehabilitation unit. Patients in the EMG-BF Group were able to receive auditory and visual feedback from the screen during the exercise.

#### Sham EMG-BF program

Patients in the Sham Group received EMG-BF with surface EMG electrodes attached to the perineal region in the hospital’s pelvic rehabilitation unit. However, the device was muted during the application and the device was positioned so that the patient could not see the screen. The screen was monitored by the physiotherapist, but the patient was not given any feedback. Only when the physiotherapist noticed that the patient had stopped exercising completely did the physiotherapist ask the patient to continue exercising.

#### PFMT

All patients participating in the study were taught PFMT. PFMT exercises were demonstrated after group assignment and before starting group-specific treatments. At the beginning of the exercise, patients were asked to quickly contract the pelvic floor two or three times, then hold it tight for 8–10 s and perform slow contractions, then rest for 10 s. Patients were informed that they should do the exercises in a sitting, lying and standing position, 3 times a day, with15 repetitions each. The EMG-BF was not used while teaching the exercises to patients. Only the pelvic floor was observed by the physiotherapist; hence, all patients were taught correct muscle contraction of the pelvic floor. Patients were given an exercise tracking chart and asked to mark the days and times they exercised. All patients brought these charts when they came for assessment.

Both EMG-BF and Sham EMG-BF applications were applied 3 days a week for 8 weeks, for a total of 24 sessions. Patients in both groups were trained with the same device (Locum Wireless Uroning system) located in the pelvic rehabilitation unit of the physical therapy hospital. The exercise duration was determined as 10 min in the first 2 weeks, 15 min in weeks 2–4, and 20 min in weeks 4–8. Patients in both the EMG-BF and Sham groups exercised in accordance with this duration. All interventions in this study were performed by the same physiotherapist (Y) experienced in pelvic rehabilitation. Participants in both groups were given additional time at the end of the program for each exercise day they could not attend due to menstruation.

### Outcome Measurements

#### Measurement of pelvic floor muscle activation with EMG

Pelvic floor muscle *activation* measurements of all patients participating in the study were performed using the NeuroTrac^®^ Simplex device. The device that performed the EMG measurements and the device that performed the EMG biofeedback application were different from each other. Therefore, it was aimed that all patients would have similar compliance with the measurement device during the study. The working average and highest, percentage of muscle used during workout, resting highest, percentage of muscle used during relaxation, working average and highest 2 s, percentage of muscle used during workout 2 s, resting average and highest 2 s, and percentage of muscle used during resting 2 s values ​​measured with the EMG device were recorded.

#### Pad Test

Objectively assesses the presence and severity of urinary incontinence. In this study, a one-hour pad test standardized by ICS was applied. Patients took 500 mL of sodium-free oral fluid over a period of 15 min. Then, a pad of known weight was given. During this time, the patient was asked to walk, go up and down stairs, sit 10 times, cough 10 times, run for 1 min, bend 5 times and wash their hands for 1 min. At the end of 1 h, the patient’s pad was weighed and the net weight was calculated.

#### The king’s health questionnaire (KHQ)

The KHQ contains a total of 32 items and is used to assess the quality of life of patients with incontinence. The KHQ scale consists of two sections. The first section focuses on general health status and quality of life. The second section evaluates urinary symptoms and their severity. High scores on the scale indicate impaired quality of life [16]. The KHQ has been culturally adapted [17].

#### International incontinence inquiry form (ICIQ-SF)

ICIQ-SF is a scale that evaluates urinary incontinence in four dimensions. The scale evaluates the frequency, amount of urinary incontinence, the impact on daily life, and the conditions that cause urinary incontinence [18]. Cultural adaptation has been made [19].

In the pelvic rehabilitation unit of the physical therapy hospital., measurements were made at the beginning, at Week 8 and at Week 20 by a physiatrist (X) who was unaware of the groups All patients’ home exercise schedules were checked when they came for measurement. Patients who did not fill at least 75% of the exercise schedule filled were excluded from the study. The exercise tracking chart was monitored by the same investigator. Menstrual periods were also marked on the exercise tracking chart and subsequently tolerated.

## Results

**Fig. 2:**
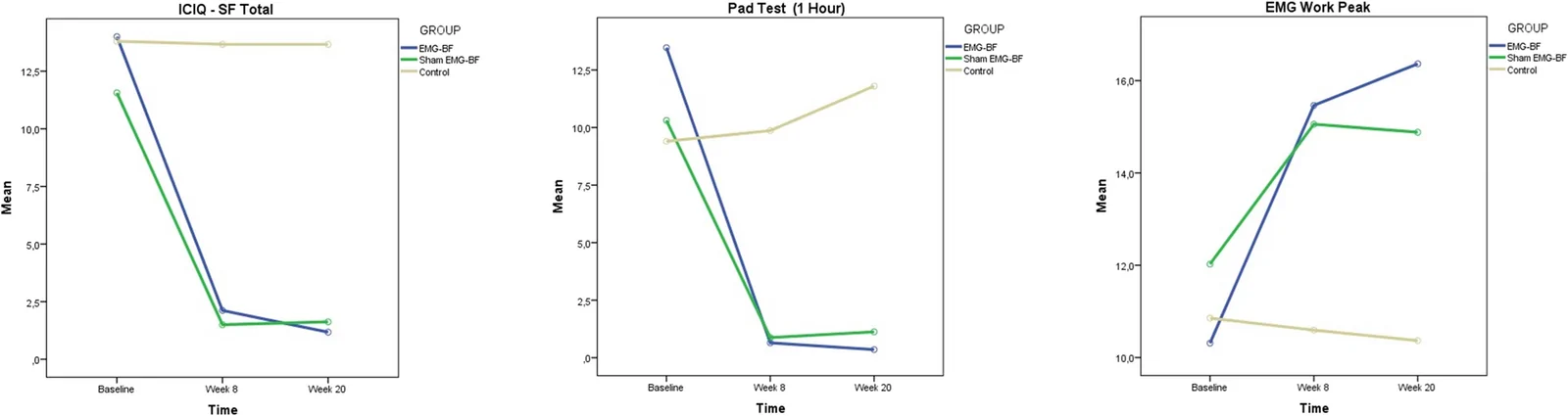
Changes in outcome measures in repeated measurements

There was no difference between the groups in terms of age, menopause duration, body mass index, and symptom duration. (Table 1) In intragroup measurements, no significant improvement was found in the control group in all parameters of ICQ-SF, while significant improvement was found in the EMG-BF and Sham EMG-BF groups. (< 0.001 for each) In intergroup measurements, EMG-BF and Sham EMG-BF groups showed statistically significant improvement in all parameters of ICQ-SF compared to the control group both at Week 8 and Week 20 (*p* < 0.001). In the Pad test, significant improvement was found in all groups in intragroup evaluation (*p* < 0.001), while in intergroup measurements, EMG-BF and Sham EMG-BF groups showed significant improvement compared to the control group both at Week 8and Week 20 (*p* < 0.001). (Table 2) (Fig. 2).

**Table 1:** Baseline parameters of the participants

Variable	EMG-BF (*n* = 17)	SHAM EMG-BF (*n* = 16)	CONTROL (*n* = 15)	*p* value
Age (mean ± SD)	47,4 ± 10,9	44,4 ± 7,4	41,1 ± 10,2	0,12
BMI (mean ± SD)	28,3 ± 4	29,1 ± 4,5	28,3 ± 6	0,84**
Menopause Duration (months) (mean ± SD)	9,8 ± 9,4	8,7 ± 6,1	12 ± 7,3	0,73**
Symptom Duration (year) (mean ± SD)	7,8 ± 8,1	6,7 ± 4,3	6,07 ± 8,8	0,79**

*BMI* Body Mass Index; **: ANOVA

**Table 2:** Comparison of Differences in Measurements by Groups and Difference Analysis Findings on ICIQ-SF and Ped Test in Repeated Measurement

Variables	Group	Before Treatment^1^ Mean ± SD	*p* value	Week 8^2^ Mean ± SD	*p* value	Week 20^3^ Mean ± SD	*p* value	*p* value	Difference post hoc** *p*
ICIQ-SF How often do you leak urine?	EMG-BF (*n* = 17)	3,41 ± 1,2	0,65*	0,53 ± 0,6	< 0,001*	0,5 ± 0,6	< 0,001*	< 0,001**	< 0,001^1,2^ < 0,001^1,3^
	Sham EMG-BF (*n* = 16)	3 ± 1,2		0,3 ± 0,6		0,3 ± 0,6		< 0,001**	< 0,001^1,2^ < 0,001^1,3^
	Control (*n* = 15)	3,2 ± 1,2		3,2 ± 1,2		3,2 ± 1,2		0,999**	-
Different Group (s)		-		EMG-BF- Control: *p* < 0,001		EMG-BF-Control; *p* < 0,001			
				Sham EMG-BF- Control: *p* < 0,001		Sham EMG-BF-Control; *p* < 0,001			
ICIQ-SF How much urine do you usually leak	EMG-BF (*n* = 17)	3,8 ± 1,7	0,46*	0,8 ± 1,01	< 0,001*	0,3 ± 0,7	< 0,001*	< 0,001**	< 0,001^1,2^ < 0,001^1,3^
	Sham EMG-BF (*n* = 16)	3,2 ± 1,4		0,7 ± 1,2		0,7 ± 1,2		< 0,001**	< 0,001^1,2^ < 0,001^1,3^
	Control (*n* = 15)	3,2 ± 1,4		3,07 ± 1,4		3,07 ± 1,4		0,36	-
Different Group(s)		-		EMG-BF-Control: *p* < 0,001		EMG-BF-Control: *p* < 0,001			
				Sham EMG-BF-Control: *p* < 0,001		Sham EMG-BF-Control: *p* < 0,001			
ICIQ-SF How much does leaking urine interfere with your everyday life?	EMG-BF (*n* = 17)	6,7 ± 2,2	0,21*	0,7 ± 1,2	< 0,001*	0,2 ± 0,9	< 0,001*	< 0,001**	< 0,001^1,2^ < 0,001^1,3^
	Sham EMG-BF (*n* = 16)	5,5 ± 2,3		0,5 ± 0,9		0,7 ± 1,1		< 0,001**	< 0,001^1,2^ < 0,001^1,3^
	Control (*n* = 15)	7,07 ± 1,1		7,07 ± 1,1		7,07 ± 1,1		0,999**	-
Different Group(s)		-		EMG-BF-Control: *p* < 0,001		EMG-BF-Control: *p* < 0,001			
				Sham EMG-BF- Control: *p* < 0,001		Sham EMG-BF-Control: *p* < 0,001			
ICIQ-SF Total	EMG-BF (*n* = 17)	14 ± 4,4	0,292*	2,12 ± 2,6	< 0,001*	1,18 ± 2,03	< 0,001*	< 0,001**	< 0,001^1,2^ < 0,001^1,3^
	Sham EMG-BF (*n* = 16)	11,56 ± 4,3		1,5 ± 2,4		1,6 ± 2,5		< 0,001**	< 0,001^1,2^ < 0,001^1,3^
	Control (*n* = 15)	13,8 ± 3,2		13,67 ± 3,2		13,6 ± 3,2		0,368	-
Different Group(s)		-		EMG-BF- Control: *p* < 0,001		EMG-BF-Control: *p* < 0,001			
				Sham EMG-BF -Control: *p* < 0,001		Sham EMG-BF-Control: *p* < 0,001			
Pad Test (1 h)	EMG-BF (*n* = 17)	13,4 ± 11,7	0,882*	0,6 ± 1,9	< 0,001*	0,3 ± 0,8	< 0,001*	< 0,001**	< 0,001^1,2^ < 0,001^1,3^
	Sham EMG-BF (*n* = 16)	10,3 ± 6,7		0,8 ± 1,8		1,1 ± 1,7		< 0,001**	< 0,001^1,2^ < 0,001^1,3^
	Control (*n* = 15)	9,4 ± 4,8		9,8 ± 4,2		11,8 ± 4,9		< 0,001**	= 0,001^1,2^ = 0,001^1,3^

*ICIQ-SF* International Incontinence Inquiry Form

In intragroup measurements, significant improvement was observed in EMG working average, Percentage of muscle used during EMG workout, EMG percentage of muscle used during relaxation, EMG working average and highest 2 s, Percentage of muscle used during EMG resting 2 s and the values ​​in EMG-BF and Sham EMG-BF groups (*p* < 0.05), while no significant improvement was observed in the control group. In intergroup measurements, significant improvement was observed in EMG-BF and Sham EMG-BF groups compared with the control group both in Week 8and Week 20 (*p* < 0.05). In intragroup measurements, significant improvement was observed in all three groups in the EMG working highest and resting highest values ​​(*p* < 0.05), while no difference was observed in intergroup measurements. (Table 3) (Fig. 2).

**Table 3:** Comparison of Differences in Measurements by Groups and Difference Analysis Findings on EMG Parameters in Repeated Measurements

Variables	Group	Before Treatment^1^ Mean ± SD	*P* value	Week 8^2^ Mean ± SD	*P* value	Week 20^3^ Mean ± SD	*P* value	*P* value	Difference post hoc** *p*
EMG working average	EMG-BF (*n* = 17)	4,4 ± 1,5	0,10*	7,6 ± 2,1	0,001*	8,3 ± 1,6	< 0,001*	< 0,001**	= 0,002^1,2^ < 0,001^1,3^
	Sham EMG-BF (*n* = 16)	5,5 ± 1,6		6,8 ± 2,3		6,8 ± 2,3		< 0,001**	= 0,002^1,2^ < 0,001^1,3^
	Control (*n* = 15)	4,7 ± 1,06		4,8 ± 1		4,6 ± 0,9		0,701**	-
Different Group(s)		-		EMG-BF- Control: *p* = 0,001		EMG-BF-Control: *p* < 0,001			
				Sham EMG-BF-Control: *p* = 0,019		Sham EMG-BF-Control: *p* = 0,009			
EMG working highest	EMG-BF (*n* = 17)	10,3 ± 4,1	0,30*	15,4647 ± 3,357	< 0,001*	16,3 ± 3,04	< 0,001*	< 0,001**	= 0,001^1,2^ < 0,001^1,3^
	Sham EMG-BF (*n* = 16)	12,0 ± 3,8		15,0563 ± 4,227		14,8 ± 4,4		0,003**	= 0,014^1,2^ = 0,008^1,3^
	Control (*n* = 15)	10,8 ± 3,8		10,593 ± 4,227		10,3 ± 4,4		0,007**	= 0,008^1,3^
Different Group(s)		-		EMG-BF-Control: *p* < 0,001		EMG-BF-Control: *p* < 0,001			
				Sham EMG-BF-Control: *p* = 0,001		Sham EMG-BF-Control: *p* = 0,004			
Percentage of muscle used during EMG workout	EMG-BF (*n* = 17)	44 ± 5,9	0,32*	49,8118 ± 7,814	0,001*	51,4 ± 6,08	< 0,001*	0,01**	= 0,01^1,3^
	Sham EMG-BF (*n* = 16)	44 ± 8,7		49,8 ± 6,849		48,5 ± 5,6		0,02**	= 0,02^1,3^
	Control (*n* = 15)	41 ± 6,6		41,78 ± 4,291		41,3 ± 4,3		0,54**	
Different Group(s)		-		EMG-BF-Control: *p* = 0,003		EMG-BF–Control: *p* < 0,001			
				Sham EMG-BF-Control: *p* = 0,004		Sham EMG-BF–Control: *p* = 0,007			
EMG resting highest	EMG-BF (*n* = 17)	7,4 ± 2,5	0,34*	11,1 ± 3	0,538*	11,5 ± 2,9	0,176*	< 0,001**	= 0,018^1,2^ < 0,001^1,3^
	Sham EMG-BF (*n* = 16)	8,8 ± 2,9		10,8 ± 4,3		10,1 ± 3,8		0,002**	= 0,011^1,2^ = 0,004^1,3^
	Control (*n* = 15)	8,9 ± 2,9		10,4 ± 4,3		9,9 ± 3,8		0,01**	= 0,032^1,2^ = 0,024^1,3^
Different Group(s)		-		-		-			
EMG percentage of muscle used during relaxation	EMG-BF (*n* = 17)	20,3 ± 7	0,075*	14,9 ± 8,5	0,029*	15,3 ± 8,9	0,158*		= 0,003^1,2^ = 0,049^1,3^
	Sham EMG-BF (*n* = 16)	17,08 ± 4,4		11,6 ± 3,6		12,9 ± 2,8			= 0,003^1,3^
	Control (*n* = 15)	15,7 ± 4,5		15,7 ± 4,4		16,3 ± 4,7			--
Different Group(s)		-		Sham EMG-BF- Control: *p* = 0,024		-			
EMG working average 2 s	EMG-BF (*n* = 17)	4,7 ± 2	0,412*	7,4 ± 2,9	0,002*	± 3,02	< 0,001*	< 0,001**	< 0,001^1,2^ < 0,001^1,3^
	Sham EMG-BF (*n* = 16)	5,4 ± 2,2		7,1 ± 1,8		7,1 ± 1,8		0,003**	= 0,024^1,2^ = 0,004^1,3^
	Control (*n* = 15)	4,9 ± 1,4		4,8 ± 1,3		4,6 ± 1,2		0,076**	-
Different Group(s)		-		EMG-BF-Control: *p* = 0,003		EMG-BF-Control: *p* < 0,001			
				Sham EMG-BF-Control: *p* = 0,004		Sham EMG-BF-Control: *p* = 0,007			
EMG working highest 2 s	EMG-BF (*n* = 17)	7,9 ± 3,8	0,356*	12,1471 ± 4,553	0,004*	12,7 ± 4,6	0,035*	< 0,001**	= 0,006^1,2^ < 0,001^1,3^
	Sham EMG-BF (*n* = 16)	9,4 ± 3,5		12,675 ± 4,254		11,3 ± 5,06		0,001**	< 0,001^1,3^
	Control (*n* = 15)	8,2 ± 3,04		8,0733 ± 2,957		8,4 ± 3,07		0,038**	-
Different Group(s)		-		EMG-BF-Control: *p* = 0,036		EMG-BF-Control: *p* = 0,033			
				Sham EMG-BF-Control: *p* = 0,005					
Percentage of muscle used during EMG workout 2 s	EMG-BF (*n* = 17)	57,2 ± 7,3	0,073*	57,8 ± 8	0,052*	57,8 ± 8,2	0,004*	0,838**	-
	Sham EMG-BF (*n* = 16)	50 ± 10,8		59,4 ± 5,8		57,4 ± 4,5		0,005**	< 0,004^1,3^
	Control (*n* = 15)	51,4 ± 9,4		51,753 ± 9,078		49,9 ± 7,6		0,074**	-
Different Group(s)		-		-		EMG-BF–Control: *p* = 0,004			
EMG resting average 2 s	EMG-BF (*n* = 17)	1,7 ± 0,6	0,194*	1,9 ± 1,8	0,341*	1,9 ± 1,4	0,096*	0,129**	-
	Sham EMG-BF (*n* = 16)	1,8 ± 0,3		1,5 ± 0,2		1,5 ± 0,2		0,007**	= 0,024^1,2^ = 0,024^1,3^
	Control (*n* = 15)	1,5 ± 0,3		1,6 ± 0,3		1,7 ± 0,2		0,008**	= 0,001^1,3^
Different Group(s)		-		-		-			
EMG resting higest 2 s	EMG-BF (*n* = 17)	7,1 ± 2,5	0,146*	10,9 ± 4,1	0,04*	11,6 ± 4,3	0,002*	< 0,001**	= 0,002^1,2^ < 0,001^1,3^
	Sham EMG-BF (*n* = 16)	9,3 ± 3,7		9,8 ± 2,6		9,5 ± 1,9		0,672**	-
	Control (*n* = 15)	7,8 ± 2,6		7,5 ± 2,3		6,9 ± 2		0,052**	
Different Group(s)		-		EMG-BF–Control: *p* = 0,042		EMG-BF-Control: *p* = 0,002			
						Sham EMG-BF-Control: *p* = 0,021			
Percentage of muscle used during EMG resting 2 s	EMG-BF (*n* = 17)	24 ± 9,5	0,17*	16,5 ± 10,3	0,036*	15,6 ± 9,4	0,082*	< 0,001**	= 0,002^1,2^ < 0,001^1,3^
	Sham EMG-BF (*n* = 16)	18,8 ± 7,2		13,3 ± 4,7		13,5 ± 3,9		0,001**	= 0,008^1,2^ = 0,002^1,3^
	Control (*n* = 15)	12,2 ± 6,4		18,7 ± 6,249		18,9 ± 7,3		0,321**	-
Different Group(s)				Sham EMG-BF-Control: *p* = 0,038					

In KHQ Emotional Problems, Sleep Energy disturbances, Symptom severity and Incontinence severity measures, significant improvement was found in all three groups in intragroup measurements (*p* < 0.05). In both the 8th week and 20th week measurements, significant improvement was found in EMG-BF and Sham EMG-BF group compared to the control group (*p* < 0.001). In KHQ General health perception, Incontinence impact, Role Limitation, Physical Limitation, Social Limitation, Personal Relationship values, significant improvement was found in EMG-BF and Sham EMG-BF groups in intragroup measurements (*p* < 0.05), while no significant improvement was found in the control group. In these values, significant improvement was recorded in EMG-BF and Sham EMG-BF groups compared to the control group both in the 8th week and 20th week (*p* < 0,01). (Table 4)

**Table 4:** Comparison of Differences in Measurements by Groups and Difference Analysis Findings on KHQ Parameters in Repeated Measurements

Variables	Group	Before Treatment^1^ Mean ± SD	*P* value	Week 8^2^ Mean ± SD	*P* value	Week 20^3^ Mean ± SD	*P* value	*P* value	Difference post hoc** * p*
KHQ-General health perception	EMG-BF (n=17)	42,6 ± 17,1	0,091**	26,4 ± 6	0,693**	25*	0,464**	<0,001**	=0,03^1,3^
	Sham EMG-BF (n=16)	34,3 ± 12,5		25 ± 12,9		25 ± 9,1		0,007***	=0,027^1,3^
	Control (n=15)	30 ± 14		28,3 ± 12,9		28,3± 12,9		0,368**	-
Different Group(s)		-		-		-			
KHQ-Incontinence impact	EMG-BF (n=17)	76,4 ± 19,5	0,240**	7,8 ± 14,5	<0,001**	1,9 ± 8	<0,001**	<0,001**	<0,001^1,2^ <0,001^1,3^
	Sham EMG-BF (n=16)	66,6± 24,3		8,3 ± 14,9		8,3 ± 14,9		<0,001**	<0,001^1,2^ <0,001^1,3^
	Control (n=15)	64,4 ± 19,7		64,4 ± 19,7		64,4 ± 19,7		0,999**	-
Different Group(s)		-		EMG-BF-Control: p<0,001		EMG-BF-Control: p<0,001			
				Sham EMG-BF – Control: p<0,001		Sham EMG-BF-Control: p<0,001			
KHQ-Role Limitation	EMG-BF (n=17)	51,9± 22,7	0,461**	5,8 ± 14,3	<0,001**	0*	<0,001**	<0,001**	<0,001^1,2^ <0,001^1,3^
	Sham EMG-BF (n=16)	44,7 ± 30,2		3,1 ± 9		1 ± 4,1		<0,001**	=0,001^1,2^ <0,001^1,3^
	Control (n=15)	55,5± 21,5		54,4± 19,3		54,4 ± 19,3		0,368**	-
Different Group(s)		-		EMG-BF-Control: p<0,001		EMG-BF-Control: p<0,001			
				Sham EMG-BF-Control: p<0,001		Sham EMG-BF-Control: p<0,001			
KHQ-Physical Limitation	EMG-BF (n=17)	60,7± 29,4	0,13**	5,8 ± 8,2	<0,001**	0*	<0,001**	<0,001**	=0,001^1,2^ <0,001^1,3^
	Sham EMG-BF (n=16)	51 ± 32,4		4,167 ± 9,6		0*		<0,001**	=0,004^1,2^ <0,001^1,3^
	Control (n=15)	74,4 ± 18,7		76,667 ± 17,5		76,667 ± 17,593		0,368**	-
Different Group(s)		-		EMG-BF-Control: p<0,001		EMG-BF-Control: p<0,001			
				Sham EMG-BF-Control: p<0,001		Sham EMG-BF -Control: p<0,001			
KHQ-Social Limitation	EMG-BF (n=17)	39,8± 28	0,001**	3,2 ± 8,5	<0,001**	0*	<0,001**	<0,001**	<0,001^1,2^ <0,001^1,3^
	Sham EMG-BF (n=16)	27,0 ± 28,3		2,7 ± 6,4		0*		<0,001**	=0,04^1,2^ =0,006^1,3^
	Control (n=15)	63,7± 12,9		64,4 ± 12,7		64,444 ± 12,738		0,999**	-
Different Group(s)		EMG-BF-Control: p=0,034		EMG-BF-Control: p<0,001		EMG-BF-Control: p<0,001			
		Sham EMG-BF-Control: p=0,001		Sham EMG-BF- Control: p<0,001		Sham EMG-BF-Control: p<0,001			
KHQ-Personal Relationship	EMG-BF (n=17)	36,2 ± 40	0,006**	5,8 ± 13	<0,001**	0*	<0,001**	0,001**	=0,039^1,3^
	Sham EMG-BF (n=16)	27± 25		2± 8,3		0*		<0,001**	=0,031^1,2^ =0,018^1,3^
	Control (n=15)	61,1± 16,2		62,2 ± 16		62,222 ± 16,019		0,867**	-
Different Group(s)		Sham EMG-BF- Control; p=0,006		EMG-Control: p<0,001		EMG-BF-Control; p<0,001			
				Sham EMG-BF-Control: p<0,001		Sham EMG-BF-Control: p<0,001			
KHQ-Emotional Problems	EMG-BF (n=17)	58,8 ± 29,8	0,006**	3,2 ± 10,9	<0,001**	0*	<0,001**	<0,001**	<0,001^1,2^ <0,001^1,3^
	Sham EMG-BF (n=16)	34,7 ± 31,1		2,7 ± 8,6		0*		<0,001**	=0,003^1,2^ =0,001^1,3^
	Control (n=15)	64,4 ± 16,3		71,8 ± 13,1		71,8 ± 13,1		0,019***	=0,019^1,2^ =0,019^1,3^
Different Group(s)		Sham EMG-BF- Control; p=0,007		EMG-BF-Control; p<0,001		EMG-BF-Control: p<0,001			
				Sham EMG-BF-Control; p<0,001		Sham EMG-BF-Control: p<0,001			
KHQ-Sleep Energy disturbances	EMG-BF (n=17)	32,3 ± 33	0,002**	0,980 ± 4	<0,001**	0*	<0,001**	<0,001**	=0,011^1,2^ =0,018^1,3^
	Sham EMG-BF (n=16)	35,4± 24,2		4,1 ± 7,4		0*		<0,001**	=0,014^1,2^ =0,001^1,3^
	Control (n=15)	63,3 ± 12,2		70± 12,2		70± 12,2		=0,009***	=0,026^1,2^ =0,026^1,3^
Different Group(s)		EMG-BF-Control: p=0,005		EMG-BF- Control: p<0,001		EMG-BF-Control: p<0,001			
		Sham EMG-BF-Control: p=0,01		Sham EMG-BF- Control: p<0,001		Sham EMG-BF-Control: p<0,001			
KHQ-Incontinence severity measures	EMG-BF (n=17)	58,4 ± 23,5	0,019**	9± 14,5	<0,001**	0,7 ± 3,2	<0,001**	<0,001**	=0,001^1,2^ <0,001^1,3^
	Sham EMG-BF (n=16)	42± 22,1		3,7 ± 5,9		0*		<0,001**	=0,003^1,2^ <0,001^1,3^
	Control (n=15)	64± 12,7		69,7± 11,5		69,77 ± 11,5		0,007***	=0,021^1,2^ =0,021^1,3^
Different Group(s)		Sham EMG-BF- Control; p=0,016		EMG-BF -Control: p<0,001		EMG-BF-Control: p<0,001			
				Sham EMG-BF-Control: p<0,001		Sham EMG-BF-Control: p<0,001			
KHQ-Symptom severity	EMG-BF (n=17)	58,3± 25,1	0,023**	10,7 ± 16,3	<0,001**	0,4 ± 2	<0,001**	<0,001**	=0,001^1,2^ <0,001^1,3^
	Sham EMG-BF (n=16)	41,6 ± 23,5		4,6 ± 7,4		0*		<0,001**	=0,003^1,2^ <0,001^1,3^
	Control (n=15)	63,8 ± 13,2		70,5± 11,2		70,5± 11,2		0,005***	=0,016^1,2^ =0,016^1,3^
Different Group(s)		Sham EMG-BF- Control; p=0,021		EMG-BF -Control: p<0,001		EMG-BF- Control; p<0,001			
				Sham EMG-BF – Control: p<0,001		Sham EMG-BF-Control; p<0,001			

*KHQ* King’s Health Questionnaire *: Kruskal Wallis; **: Friedman ***:Repeated Measures ANOVA, ****: Wilcoxon Testi; *: Since the observation values ​​are the same, the standard deviation is equal to 0

## Discussion

In this study, adding BF treatment to PFMT exercises is effective in improving the frequency of urinary incontinence, quality of life, pelvic floor muscle usage percentage and muscle *activation* effective during 2-second contraction. This effectiveness was achieved with both the EMG-BF application and sham applications. When applied alone, PFMT exercises are effective on sleep disturbances, emotional problems, incontinence duration, frequency and pad test due to urinary incontinence. However, when EMG-BF application is added to PFMT, its effectiveness increases. This effectiveness was achieved with both the EMG-BF application and sham application.

Additionally, PFMT had no effect on general health perception, role limitations due to incontinence, physical and social limitations, and personal relationships. Adding EMG-BF treatment to PFMT exercises results in improvements in these parameters, and furthermore, sham EMG-BF application is as effective as real EMG-BF application. According to the results of this study, EMG-BF exercise performed under the supervision of a physiotherapist is an effective method for women with SUI. Auditory and visual feedback provided by EMG-BF has no additional contribution to pelvic floor muscle strengthening. The reason for EMG-BF effectiveness may be due to the patient learning and repeating the use of the pelvic floor muscles. In this study, applying pelvic floor muscle training under the supervision of a physiotherapist for a certain period of time and with repetition provided the same effect even without visual and auditory feedback. According to the authors, this effectiveness was possible with the patient’s high motivation and participation and ability to maintain appropriate muscle control. To the best of the investigators’ knowledge, this is the first study to evaluate the effectiveness of EMG-BF using sham application in the treatment of urinary incontinence. In the literature, the effectiveness of EMG-BF has been investigated using sham application in neurological rehabilitation, but no study has been found examining the effectiveness of EMG-BF using sham application in pelvic rehabilitation [13, 22].

In this study, while PFMT exercises performed at home did not have an effect on general health perception, role limitations due to incontinence, physical and social limitations and personal relationships, improvement with exercises performed under the supervision of a physiotherapist in a hospital environment might be attributed to the biopsychosocial aspect of incontinence. Receiving supervised physiotherapy in the hospital both improved pelvic floor muscle *activation* and increased the patient’s participation in treatment and social life. The OPAL RCT recommended that studies should be planned for pelvic floor rehabilitation, especially those involving healthcare personnel. In the mentioned study, EMG-BF was applied as a home program and EMG-BF effectiveness could not be demonstrated [9]. Ibrahim et al. compared the effectiveness of EMG-BF treatment with PFMT in the study they conducted in the EMG-BF unit in the hospital. As a result of the study, they reported that EMG-BF was effective in improving electrophysiological measurements and pelvic muscle contraction, quality of life and urinary incontinence. They also emphasized that patient participation and compliance were better with EMG-BF [23]. Sahin et al. compared the effectiveness of EMG-BF with vaginal cone in patients with SUI. They did not use a control group in the study, followed the patients for 6 months and reported the effectiveness on muscle strength, quality of life and symptoms in the EMG-BF group [22]. The findings of this study also support existing literature. In another study, EMG-BF effectiveness was compared with Pilates and no control group was used. While EMG-BF was effective on incontinence symptoms and muscle activity, pilates was reported to be more effective on quality of life compared to EMG-BF [10].

This study was conducted with women living in a rural area. Additionally, we excluded individuals who exercise more than 2 h per week in order to achieve homogenization. Although PFMT was taught by an experienced physiotherapist, the patient population of this study may have had lower muscle awareness and difficulty complying with active home exercise therapy. The authors think that the lower treatment success in patients receiving only PFMT may be related to the patient population of this study.

### Limitations

In this study, although there was no difference between the three groups in terms of menopause duration, menopause was not included among the exclusion or inclusion criterion. This heterogeneity is a limitation that should be taken into consideration when examining the study data. When evaluating the data from this study, it should be kept in mind that cross-interaction problems in the perineal surface electrodes due to other muscles. Furthermore, in this study, all applications were performed in a hospital setting under the supervision of a physiotherapist. This should be taken into consideration when interpreting the results. We recommend that future studies examine the difference between performing EMG BF at home and in a hospital setting with a physiotherapist. However, the strengths of this study are its blind design using a control group and a sham group, the involvement of a specialized pelvic rehabilitation team, and the relatively long follow-up period.

## Conclusion

Adding EMG-BF therapy to PFMT exercises is effective on pelvic floor muscle *activation* h, quality of life, frequency and amount of urinary incontinence. Additionally, PFMT exercises alone did not have an effect on general health perception, role limitations due to incontinence, physical and social limitations, and personal relationships either. Adding EMG-BF application to the treatment improved these parameters. EMG-BF exercises performed under the supervision of a physiotherapist are an effective method even without auditory and visual feedback.

### Ethical Approval

The study was conducted in accordance with the Declaration of Helsinki. Informed consent was obtained from all study participants before starting the study. Before starting the study, approval was received from the University’s Clinical Research Ethics Committee with the number XXX. After ethics committee approval, the study was registered on clinicaltrials.gov before patients were recruited into the study. (Clinical Trial NCTXXX) This study was designed and conducted in accordance with CONSORT guidelines [20]. 

### Sample Size Calculation

G-Power 3.1.9.6 program was used to calculate the sample size of the study. By making use of the findings of similar studies in the literature, it was decided that a minimum of 36 people in total, 12 in each group, should be studied with 80% power and a 5% margin of error in order to achieve a medium level effect size (d = 0.46) between dependent measurements [21]. However, since there were more patient applications during the planned period of the study, the study was conducted with 60 patients. After the study was completed, post-hoc power analysis was performed using the G-Power 3.1.9.4 program. In group comparisons, the power for measurements of parameters with *p* < 0.05/group comparisons was calculated as over 80%.

### Statistical Analysis

Mean ± Standard Deviation (SD); median, minimum, maximum and frequency (n) and percentage (%) values were reported for numerical and categorical variables, respectively. The normality assumption was tested via the Shapiro-Wilk Test. Two-Way Repeated Measures ANOVA was applied fort the parameters. The Interaction Term (Time*Group) was observed to be statistically significant for the parameters (*p* < 0.05, for all). The Kruskal-Wallis (K-W) Test was utilized for the group comparisons at each time-point, namely before treatment, after treatment, and 3-months after the treatment. The Dunn Test was used for pairwise comparisons. Friedman Test, Repeated Measures ANOVA and Wilcoxon Test were performed to determine whether there was a significant difference between dependent measures within each group (EMG, Placebo, Control). Bonferroni correction was applied in identifying the different measures. A two-sided p-value ≤ 0.05 was taken as the threshold for statistically significant. R Programming Language (R Core Team (2023), R Foundation for Statistical Computing, Vienna, Austria) was used for all the analyses.

## Supplementary Information

Supplementary Material 1 (XLSX 13.9 KB)

## Data Availability

Data supporting the findings of this study are available from the corresponding author upon reasonable request.

## Declarations

### Permission to Reproduce Material From other Sources

This study does not include any material reproduced from other sources
